# Respiratory diseases and the gut microbiota: an updated review

**DOI:** 10.3389/fcimb.2025.1629005

**Published:** 2025-08-11

**Authors:** Xin Yu, Xiao Yu, Yubo Wang, Xiaoping Guo, Chao Wang, Fang Wang

**Affiliations:** ^1^ Department of Pathogen Biology, College of Basic Medical Sciences, Jilin University, Changchun, China; ^2^ Department of Histology and Embryology, College of Basic Medical Sciences, Jilin University, Changchun, China; ^3^ Department of Laboratory Medicine, China-Japan Union Hospital of Jilin University, Changchun, China; ^4^ The Medical Basic Research Innovation Center of Airway Disease in North China, Ministry of Education, Changchun, China; ^5^ Jilin Provincial Key Laboratory of Precision Infectious Diseases, Jilin University, Changchun, China; ^6^ Jilin Provincial Engineering Laboratory of Precision Prevention and Control for Common Diseases, Jilin University, Changchun, China

**Keywords:** gut microbiota, respiratory diseases, gut-lung axis, short-chain fatty acids, fecal microbiota transplantation, probiotics, postbiotics, dietary fibers

## Abstract

The gut microbiota constitutes a vital ecosystem within the human body playing a pivotal role in immune regulation and metabolic homeostasis. Emerging research underscores a sophisticated interplay between the gut and lungs, termed the “gut-lung axis.” Gut microbes exert influence over pulmonary immunity and metabolism via immune mediators (e.g., cytokines and interleukins), metabolites (e.g., short-chain fatty acids) and direct microbial translocation. Dysbiosis of the gut microbiota has been implicated in a spectrum of respiratory diseases, including asthma, chronic obstructive pulmonary disease (COPD), acute lung injury (ALI), Coronavirus Disease 2019 (COVID-19), lung cancer, idiopathic pulmonary fibrosis (IPF), pulmonary arterial hypertension (PAH), acute lower respiratory infection (ALRI) and tuberculosis (TB). Although multi-omics technologies have elucidated certain mechanisms underlying the gut-lung axis, numerous pathways remain to be fully delineated. This review synthesizes current knowledge on the role of gut microbiota and their metabolites in respiratory diseases and assesses their therapeutic potential. Future investigations should prioritize strategies to restore and maintain microbial homeostasis, such as dietary modifications, probiotic supplementation and fecal microbiota transplantation to pioneer novel preventive and therapeutic approaches. These summaries of advances in gut microbiology research promise better management and exploration of therapeutic strategies for respiratory diseases.

## 1 Introduction

The human body harbors a diverse array of microorganisms with the gut being the most densely colonized organ, hosting up to 100 trillion (10^14^) microorganisms (Ley et al., 2006; Grice and Segre, 2012; Dang and Marsland, 2019) including bacteria, fungi, and viruses (Parker et al., 2020). These gut microbiota have co-evolved with the host, establishing a complex and symbiotic relationship (Wei et al., 2024). Their relative stability is critical for maintaining the body’s immune system and metabolic balance (Bock et al., 2024; Ullah et al., 2024). Additionally, gut microbes influence systemic homeostasis by modulating metabolite levels (Canfora et al., 2019). Emerging evidence highlighted the pivotal role of gut microbiota in regulating the local immune system, particularly through the modulation of neutrophil responses and pro-inflammatory signaling pathways (Danne et al., 2024; Ye et al., 2025). Consequently, ecological dysregulation of the gut microbiota may contribute to the pathophysiology of lung diseases by disrupting immune homeostasis, impairing nutrient absorption and altering metabolic equilibrium (Chu and Mazmanian, 2013; Kamada et al., 2013; Zheng et al., 2020).

Although the lungs and the gut are anatomically distinct organs, accumulating evidence suggests a close and dynamic interaction between them (Cheng et al., 2024; Kim et al., 2024), which is essential for maintaining organismal stability (Enaud et al., 2020). This interplay has led to the proposal of a novel concept: the “ gut-lung axis” (Jia et al., 2024; Sun et al., 2024). The gut-lung axis encompasses the bidirectional communication and interaction pathways between the lungs and the gut, mediated through various mechanisms including the immune system, the nervous system and metabolic substances (Budden et al., 2017; Chiu et al., 2022). Gut microbiota play a critical role in regulating the immune system influencing not only intestinal immunity (Zhou et al., 2021) but also pulmonary immune responses through the modulation of cytokines, interleukins and other signaling molecules (McAleer and Kolls, 2018; Espírito Santo et al., 2021). These interactions can potentially trigger lung infections and inflammatory responses. For instance, gut microbial dysbiosis can lead to abnormal immune system activation, resulting in the excessive release of inflammatory mediators such as TNF-γ and IL-6 (Wu Y. et al., 2022; Wu R. et al., 2024). Furthermore, gut microorganisms produce a wide range of metabolites including short-chain fatty acids, amino acids and vitamins, which can enter systemic circulation and influence host metabolic homeostasis (Hays et al., 2024; Li TT. et al., 2024; Zhan et al., 2024). Furthermore, recent research by our research group has found that succinate produced by the gut microbiota can act on the lungs, affecting protein post-translational modifications and metabolic homeostasis in the lungs (Wang et al., 2025). This result further provides evidence for the existence of the gut-lung axis. In some cases, gut microorganisms may translocate directly to the lungs via the bloodstream or lymphatic system, potentially causing lung infections or inflammation (Dickson and Huffnagle, 2015; Schuijt et al., 2016). This mechanism provides new insights into the intricate relationship between gut microbiota and lung homeostasis, highlighting the importance of the gut-lung axis in health and disease (Felix et al., 2018).

Respiratory diseases as prevalent conditions capable of triggering systemic reactions have long been a focal point of research (Zhao et al., 2023). Notably, patients with gastrointestinal disorders often exhibit disruptions in pulmonary homeostasis, which are associated with an elevated incidence of respiratory diseases (Massart and Hunt, 2020). Recent advancements in microbiomics and metagenomics have deepened our understanding of the gut-lung axis by uncovering potential connections between gut and lung microbiota (Song et al., 2020; Boesch et al., 2021; Eladham et al., 2024; Li R. et al., 2024). This review examines the latest findings on the role of gut microbiota and their metabolites in common respiratory diseases, including chronic obstructive pulmonary disease (COPD), asthma, acute lung injury (ALI), Coronavirus Disease 2019 (COVID-19), lung cancer, idiopathic pulmonary fibrosis (IPF), pulmonary arterial hypertension (PAH), acute lower respiratory infection (ALRI), and tuberculosis (TB), as well as the underlying mechanisms. Additionally, it summarizes emerging therapeutic strategies targeting gut microbiota offering new insights into the management of respiratory diseases.

## 2 The role of gut microbes in specific respiratory diseases

**Figure 1:**
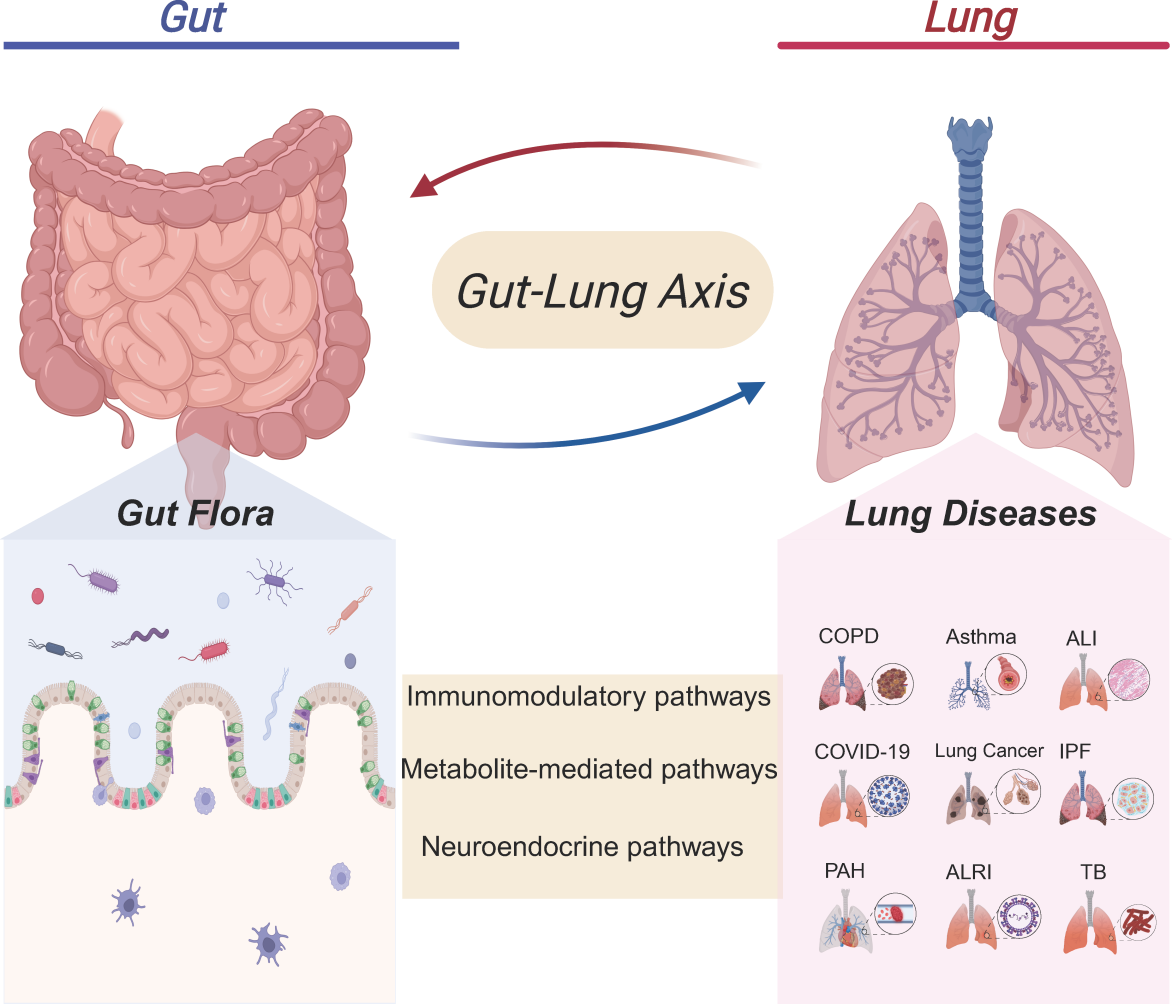
Crosstalk between the gut microbiota and pulmonary diseases. The gut microbiota in the Gut can interact with the Lung through immunomodulatory pathways, metabolite- mediated pathways, and neuroendocrine pathways. This gut - lung axis association involves multiple lung diseases such as chronic obstructive pulmonary disease (COPD), Asthma, Acute lung injury (ALI), coronavirus disease 2019 (COVID-19), Lung cancer, Idiopathic pulmonary fibrosis (IPF), Pulmonary arterial hypertension (PAH), Acute lower respiratory infection (ALRI), and Tuberculosis (TB). It suggests that the gut microbiota may affect lung health through the gut-lung axis, providing a new gut-related perspective for the research and intervention of lung diseases.

Despite the anatomical distance separating the lungs and the gut within the human body, advancements in multi-omics technologies have increasingly demonstrated a significant correlation between alterations in gut microbiota composition and the immune response of the respiratory system, as well as the maintenance of pulmonary homeostasis. Experimental studies and epidemiological data have substantiated a critical connection between the gut microbiota and lung health, conceptualized as the “gut-lung axis.” Nonetheless, the precise mechanisms and pathways underpinning this relationship warrant further elucidation. Dysbiosis is characterized by disruptions in the composition and functionality of the gut microbiota plays a pivotal role in modulating the body’s immune responses and has been implicated in the pathogenesis of various pulmonary diseases. **Table 1** presents the specific changes in the gut and lung microbiota of patients with respiratory diseases. However, the existing body of research in this domain remains limited. Future investigations are anticipated to yield more comprehensive insights into the gut-lung axis, as illustrated in **Figure 1**, offering promising avenues for exploration.

**Table 1:** Summary of specific microbial taxa involved in various respiratory diseases (clinical cohorts).

Disease	Variables	Summary findings
COPD (Chiu et al., 2022)	Stool samples	The *Firmicutes* phylum is more abundant in COPD.
COPD (Li et al., 2021)	Stool samples	In COPD, the number of *Prevotella* bacteria increases, and lung function improves after fecal microbiota transplantation.
COPD (Bowerman et al., 2020)	Stool samples	The streptococcus species has been identified as a key differentiating factor between COPD patients and healthy individuals.
Asthma (DeVries et al., 2022)	Nsal samples	At the age of 36 months, asthmatic newborns transition from early *Haemophilus* colonization to being dominated by Moraxella.
Asthma (Levan et al., 2019)	Stool samples	The elevated concentration of 12,13-diHOME in asthma can hinder immune tolerance, and it may be produced by the bacteria epoxide hydrolase in the neonatal intestinal tract.
Asthma (Arrieta et al., 2015)	Stool samples	*Lachnospira, Veillonella, Faecalibacterium* and *Rothia* were significantly reduced in children at risk of asthma. Vaccinating germ-free mice with these four bacterial groups improved the airway inflammation in their adult offspring.
Asthma (Kanj et al., 2023)	Stool samples	Disorder of the *Candida* intestinal microbiota may worsen the control of asthma.
ALI (Zeng et al., 2023)	Stool samples	The diversity of the fecal microbiota in patients with lung diseases treated with antibiotics decreases. The level of AI-2 in feces is positively correlated with inflammatory molecules in the serum of lung disease patients.
ALI (Wang YH. et al., 2023)	Stool and BALF samples	The succinate derived from the intestinal microbiota enhances the lung alveolar macrophage polarization in response to intestinal ischemia-reperfusion-induced ALI through SUCNR1-dependent mechanism.
COVID-19 (Zuo et al., 2020b)	Stool samples	The baseline abundance of *Coprobacillus*, *Clostridium ramosum*, and *Clostridium hathewayi* correlated with COVID-19 severity.
COVID-19 (Yeoh et al., 2021)	Stool samples	*Faecalibacterium prausnitzii, Eubacterium rectale* and *bifidobacteria* showed a significant decrease.
Lung cancer (Zhao et al., 2021)	Stool and Serum samples	There is an intricate relationship between gut microbiome and levels of several metabolites such as glycerophospholipids and imidazopyrimidines.
Lung cancer (Lu et al., 2023)	Stool samples	*R. gnavus* was significantly upregulated in lung cancer patients.
IPF (Göktürk et al., 2024)	Stool samples	Compared with the control group, the IPF patient groups exhibited lower abundance of *Actinobacteria*, *Bifidobacteriales*, *Burkholderiales*, *Bacteroidaceae*, *Dorea*, *Fusicatenibacter*, and *Ruminococcus gauvreauii*, while showing higher abundance of *Enterobacterales*, *Erysipelotrichaceae*, *Holdemanella*, and *Alloprevotella*
PAH (Kim et al., 2020)	Stool samples	In the group of patients with PAH, the number of bacteria that can produce butyrate and propionate (such as *Coprococcus*, *Butyrivibrio*, *Lachnospiraceae*, *Eubacterium*, *Akkermansia*, and *Bacteroides*) has decreased.
PAH (Jose et al., 2022)	Stool samples	The relative abundance of *Lachnospiraceae* bacterium GAM79 in the feces of PAH patients is lower than that in non-PAH control subjects.
PAH (Moutsoglou et al., 2023)	Stool samples	PAH patients had poor gut microbiome diversity and distinct phylogenetic characteristics, which were manifested by a reduction in anti-inflammatory metabolite-related microorganisms and their products, as well as an enrichment of pro-inflammatory metabolite-related microorganisms.
H1N1 (Gu et al., 2020)	Stool samples	The microbial diversity of H1N1 patients is reduced, and the number of several representative anaerobic butyrate-producing bacteria from the Lachnospiraceae and Ruminococcaceae families is significantly decreased.
Bronchiolitis (Cabrera-Rubio et al., 2024)	Feces and respiratory nasopharyngeal aspirate	A significant reduction in intestinal microbial richness and an increase in respiratory microbial diversity can be observed.
TB (Wang et al., 2022)	Stool samples	Dominant genera such as *Bacteroides, Parabacteroides, Fusobacterium*, and *Lachnoclostridium* were significantly enriched; in contrast, the healthy people group was enriched with Blautia, *Roseburia, Bifidobacterium*, unclassified*_Ruminococcaceae, Clostridium, and Romboutsia*
TB (Ye et al., 2022)	Stool samples	The relative abundance of *Bacteroides, Parabacteroides*, and *Veillonella* was significantly higher in the TB group, while the relative abundance of *Faecalibacterium, Bifidobacterium, Agathobacter*, and CAG-352 was significantly lower

### 2.1 COPD

COPD is clinically characterized by chronic inflammation and obstructive lung pathology (Song et al., 2023). Globally recognized risk factors for COPD include smoking and environmental pollution (Jiang et al., 2024). There is increasing evidence highlighting a significant interplay between gut microbiota composition and lung immunity. Peiju Fang et al. demonstrated that exposure to cigarette smoke and particulate matter disrupts the intestinal microbiota, suggesting that gut microbiota disturbances may contribute to COPD progression (Huang et al., 2024; Fang et al., 2025). A retrospective analysis of 1,228 COPD patients revealed that a majority exhibited at least one gastrointestinal symptom with a notably higher prevalence of inflammatory bowel disease and irritable bowel syndrome compared to the healthy population (Rutten et al., 2014). In a study involving 55 patients with COPD, it was found that at the phylum level, the *Bacteroidetes* phylum was more abundant in the control group, while the *Firmicutes* phylum was more abundant in the COPD group. At the genus level, the *Alloprevotalla* genus was more abundant in the control group than in the COPD group (Chiu et al., 2022). Naijian Li et al. demonstrated in a mouse model that alterations in the gut microbiota of COPD patients are linked to disease progression. Mice receiving fecal transplants from COPD patients exhibited significant lung pathology, reduced lung function and increased airway mucus production (Li et al., 2021). Kate L. Bowerman et al. identified distinct differences in gut microbiota composition and metabolomics between COPD patients and healthy individuals with *Streptococcus* spp. being a key differentiating factor. Certain members of this genus were strongly associated with reduced lung function (Bowerman et al., 2020). Dysbiosis in the gut microbiota leads to increased production of harmful bacterial metabolites and decreased short-chain fatty acid synthesis, exacerbating intestinal permeability and facilitating bacterial translocation. Immune cells in the gut interact with these metabolites and microbiota, resulting in the release of pro-inflammatory cytokines into the bloodstream, which further exacerbates COPD progression via the gut-lung axis (Li N. et al., 2023). Collectively, these findings suggest that disruption of the intestinal barrier may promote the translocation of dysregulated microbiota to the airways, thereby affecting pulmonary homeostasis (Giron et al., 2021; Ruan et al., 2022; Lim et al., 2023). Translocated pathogens and their virulence factors may also infiltrate the gastrointestinal tract, triggering intestinal inflammation and related disorders (Dong et al., 2024).

### 2.2 Asthma

Asthma is a chronic inflammatory disease characterized by a complex and diverse pathophysiology. Substantial research confirms that alterations in the microbiota during early life are closely associated with the onset and progression of asthma (Liu C. et al., 2022; Alhasan et al., 2023; Kahhaleh et al., 2024). The colonization of the gut microbiota is crucial for immune system development, and disruptions in this process may increase susceptibility to asthma in children (Casali and Stella, 2024; Ke et al., 2025). Studies have shown that the characteristics of newborns of mothers with asthma are that they are colonized by *Haemophilus bacteria* in the early stage of life, and at the age of 36 months, the microbial community shifts to being dominated by *Moraxella bacteria (* DeVries et al., 2022). In neonates at high risk of asthma, the concentration of 12,13-diHOME, which is produced by intestinal bacteria, is elevated in their feces (Levan et al., 2019). This substance can induce long-term immune effects, leading to CD4^+^ T cell dysfunction [59,60], thereby increasing susceptibility to asthma (Nilsen et al., 2024; Sanidad et al., 2024). As children grow, gut microbial diversity gradually increases, maturing into an adult-type microbiota composition by age three, characterized by elevated levels of *Firmicutes* and *Proteobacteria* and reduced levels of *Actinobacteria* and *Bacteroidetes (* Ximenez and Torres, 2017), Notably, asthmatic individuals exhibit increased diversity of *Aspergillus* spp (Celik et al., 2024). Polysaccharide A from *Bacteroides fragilis* has been shown to promote IL-10 secretion by CD4^+^ T cells (Zhong et al., 2024), while extracellular polysaccharides from *Bifidobacterium longum* inhibit Th17 responses in both the gut and lungs (Li D. et al., 2025), thereby reducing asthma susceptibility. The role of gut microbiome dysregulation in asthma progression has been extensively investigated in both human patients and mouse models (Smulders et al., 2024; Liu et al., 2025). Experiments involving germ-free mice have demonstrated that microbial exposure is critical for the morphological and functional development of the immune system (Anandakumar et al., 2024). Arrieta et al. revealed significant reductions in the abundance of *Lachnospira*, *Veillonella*, *E. faecalis*, and *Rothia* in the gut microbiota of infants with asthma. Inoculation of these bacteria into germ-free mice alleviated airway inflammation and prevented asthma development (Arrieta et al., 2015). Amjad N. Kanj’s study further confirmed that dysbiosis of the gut fungal microbiota, particularly involving *Candida*, enhances Th2 responses in mice sensitized with house dust mite, exacerbating asthmatic airway inflammation (Kanj et al., 2023). Collectively, these studies demonstrated that gut microorganisms influence the host immune status by modulating immune cell activity and regulating inflammatory factor production, thereby impacting the onset and progression of asthma. These findings underscored the importance of focusing on the gut-lung axis during early life and developing effective asthma prevention strategies. Examples include antibiotic use during pregnancy and infancy as well as probiotic therapies to promote gut homeostasis and reduce the likelihood of asthma development (Lee et al., 2023).

### 2.3 ALI

ALI is a severe respiratory condition triggered by various etiological factors (Ren Z. et al., 2024; Zhang et al., 2025). Disruptions in the gut microbiota can activate the systemic immune system and facilitate bacterial translocation to the lungs, thereby exacerbating the pathological progression of lung injury (Wang Q. et al., 2024). An imbalance in the gut microbiota modulates the TLR4/NF-κB signaling pathway within the lung immune system and induces oxidative stress, contributing to lung injury (Hua et al., 2023). Xianghao Zeng et al. observed reduced fecal microbiota diversity in pneumonia patients treated with antibiotics compared to healthy volunteers. They identified gut microbiota-derived autoinducer-2 (AI-2), a metabolite derived from the gut microbiota, as positively correlated with inflammatory molecules in fecal samples. Intraperitoneal administration of AI-2 in a mouse model of acute lung injury resulted in heightened lung inflammation, characterized by elevated levels of IL-6, IL-1β, and chemokines (Zeng et al., 2023). Zhengjian Wang demonstrated that modulating the gut microbiota composition in mice—by reducing the *Firmicutes*/*Bacteroidetes* ratio and increasing the relative abundance of short chain fatty acids (SCFAs)-producing bacteria—activated the AMPK/NF-κB/NLRP3 signaling pathway, offering a therapeutic approach for ALI (Wang Z. et al., 2023). Similarly, succinate as a metabolite produced by the gut microbiota was shown to exacerbate the inflammatory response in acute lung injury through succinate receptor 1 (SUCNR1)-dependent polarization of alveolar macrophages (Wang YH. et al., 2023).

Consequently, the gut microbiota has emerged as a promising therapeutic target for the treatment of ALI. For instance, Wei Song et al. demonstrated that the protective effects of cymbopogonin against septic acute lung injury are likely mediated through the regulation of gut microbiota and restoration of the intestinal barrier. The underlying mechanism appears to involve the aryl hydrocarbon receptor (AHR)/Nrf2 signaling pathway (Song et al., 2021). Additionally, acetic acid as a metabolite of the gut microbiota has been shown to mitigate septic ALI by modulating the MAPK pathway. This intervention improves alveolar permeability, reduces the levels of inflammatory factors, and suppresses the production of oxygen radicals (Xu et al., 2019). These findings collectively highlight the critical role of gut microbiota dysbiosis in the pathogenesis of ALI, emphasizing the potential for therapeutic interventions targeting gut-lung axis interactions.

### 2.4 COVID-19

COVID-19, caused by the novel severe acute respiratory syndrome coronavirus 2 (SARS-CoV-2), is primarily a respiratory illness (Zuo et al., 2020b). Previous studies have revealed a significant reduction in bacterial diversity within the fecal microbiome of COVID-19 patients (Mizutani et al., 2022; Zhang et al., 2023), alongside a decreased abundance of SCFAs-producing bacteria from the family *Lachnospiraceae (* Xu et al., 2021). Emerging evidence indicates that SARS-CoV-2 not only infects the lungs but also targets the gastrointestinal tract (Pagnini et al., 2020; Uno, 2020; Xiao et al., 2020). Following COVID-19 infection, patients exhibit distinct alterations in their gut microbiome, characterized by reduced levels of *Ruminococcus gnavus*, *Bacteroides vulgatus*, and *Faecalibacterium prausnitzii*, alongside an increase in the phyla *Firmicutes* and *Actinobacteria (* de Oliveira et al., 2021). This dysbiosis is marked by the enrichment of opportunistic pathogens and the depletion of beneficial microorganisms (Zuo et al., 2020b). Clinical data have demonstrated a negative correlation between the abundance of *Bifidobacterium adolescentis*, *Bifidobacterium bifidum*, *Bifidobacterium longum*, and *Bifidobacterium pseudocatenulatum* and SARS-CoV-2 viral loads in patients’ fecal samples (Zuo et al., 2020b; Zuo et al., 2020a; Peng et al., 2021). These findings suggest that gut microbiota alterations may play a critical role in the persistence of respiratory symptoms post-infection (Bernard-Raichon et al., 2022; Liu Q. et al., 2022). The gut microbiota may influence SARS-CoV-2 replication (Xu et al., 2021; Nagai et al., 2023) through the migration of immune cells across mucosal surfaces, interactions between the gut and lungs, and the production of cytokines on the intestinal mucosa (Nagata et al., 2023). Yun Kit Yeoh et al. analyzed fecal samples from 100 patients with confirmed SARS-CoV-2 infection and observed reduced levels of immunomodulatory bacteria such as *Eubacterium rectale*, *Faecalibacterium prausnitzii*, and *Bifidobacterium bifidum*. Concurrently, these patients exhibited elevated concentrations of inflammatory cytokines, which correlated with disease severity. This suggests that the gut microbiota may modulate the host immune response during COVID-19 infection (Yeoh et al., 2021). Furthermore, COVID-19 patients experienced significant and persistent changes in their fecal microbiome throughout hospitalization, characterized by the enrichment of opportunistic pathogens and the depletion of beneficial microbes. Notably, this dysbiosis persisted even after SARS-CoV-2 clearance (as confirmed by pharyngeal swabs) and the resolution of respiratory symptoms, highlighting the long-term impact of COVID-19 on gut microbiota composition and function.

The Syrian hamster model can recapitulate some pulmonary features of human COVID-19, and gastrointestinal changes during infection occur concomitantly with alterations in gut microbiota composition, characterized by increased relative abundance of deleterious bacterial taxa such as *Enterobacteriaceae* and *Desulfovibrionaceae*, whereas bacteria capable of producing SCFAs (e.g., *Ruminococcaceae* and *Lachnospiraceae*) show a decreased relative proportion (Sencio et al., 2022b). Findings from metagenomic and metabolomic studies demonstrated that: sustained decreases in phenylalanine, tryptophan, glutamate, and indoleacetic acid in elderly hamsters were positively correlated with poor weight recovery and/or pulmonary fibrosis during the recovery phase of SARS-CoV-2 infection; whereas in young hamsters, bacterial taxa such as *Eubacterium*, *Oscillospiraceae*, and *Lawsonibacter*, as well as plasma metabolites including carnosine and cis-aconitic acid, were all associated with mild disease outcomes (Brito Rodrigues et al., 2025). These findings indicate that developing age-specific microbiome-targeted strategies would contribute to more effective management of acute viral pneumonia and its long-term prognosis. Another study also revealed that changes in the gut microbial community were characterized by increased abundance of several genera previously linked to intestinal inflammation and disease (*Haemophilus*, *Fusobacterium*, *Streptococcus*, *Campylobacter*, and *Johnsonella*) (Seibert et al., 2022). Obese patients with non-alcoholic steatohepatitis (NASH) are highly susceptible to COVID-19. In obese NASH hamsters, certain taxa such as *Blautia* and *Peptococcus* were associated with pro-inflammatory parameters in the lung and liver; these taxonomic profiles and their associations with specific disease markers suggested that microbial patterns may influence COVID-19 outcomes (Sencio et al., 2022a). Additionally, oral administration of the gut microbes *Oribacterium* sp. *GMB0313* and *Ruminococcus* sp. *GMB0270* to hamsters conferred complete protection against SARS-CoV-2 infection by activating CD8^+^T cell-mediated immunity, with preventive efficacy comparable to or even superior to that of mRNA vaccines (Wang M. et al., 2024).

### 2.5 Lung cancer

Lung cancer remains one of the most prevalent malignant tumors globally, with its incidence and mortality rates consistently ranking among the highest for cancers (Sun et al., 2023). Microorganisms colonizing the human body can significantly influence tumor cell metabolism, growth patterns, and functions, as well as shape the tumor microenvironment (El Tekle and Garrett, 2023). Emerging evidence highlights the gut microbiota and their associated metabolites as critical factors contributing to and regulating the development of lung cancer (Zhao et al., 2021). Using Mendelian randomization analysis with the inverse variance weighted (IVW) method as the primary approach, Ying chen Li et al. identified 40 distinct groups of gut microbiota in lung cancer patients, establishing a causal relationship between specific gut microbial communities and lung cancer (Li Y. et al., 2023). Further studies have analyzed 16S rRNA gene sequencing and metabolomics data from stool and serum samples of lung cancer patients and healthy controls. These investigations revealed that specific gut microbes, such as *Clostridium difficile* and *Bacillus augmentans*, along with their associated metabolites, could serve as potential diagnostic biomarkers and therapeutic targets for lung cancer (Zhao et al., 2021). Notably, the gut microbiome composition varies among different subtypes of lung cancer. For instance, patients with squamous cell carcinoma exhibit a higher abundance of *Aspergillus*, *Gammaproteobacteria*, *Enterobacteriaceae*, and *Firmicutes*, while those with adenocarcinoma show increased levels of *Clostridium* and *Roseburia* species (Lu et al., 2023). These findings underscore the role of gut microbiota in lung cancer pathogenesis and progression. Researchers have explored the therapeutic potential of modulating the gut microbiota to influence lung cancer outcomes. For example, traditional herbal formulations such as Zeng sheng Ping have been shown to boost immunity, protect the intestinal mucosa, and regulate gut microbiota, thereby impacting lung cancer progression (Sun et al., 2023). Additionally, ginseng polysaccharides have demonstrated the ability to enhance the antitumor response to αPD-1 monoclonal antibody therapy by increasing the microbial metabolite valeric acid and reducing the ratio of L-kynurenine to tryptophan (Kyn/Trp) (Huang et al., 2022). These approaches represent innovative strategies in the prevention and treatment of lung cancer, highlighting the pivotal role of the gut-lung axis in oncology.

### 2.6 Idiopathic pulmonary fibrosis

Idiopathic pulmonary fibrosis (IPF) is the most common type of idiopathic interstitial pneumonia, accounting for approximately one-third of all interstitial lung disease cases (Maher, 2024).Compared with the control group, the IPF patient groups exhibited lower abundance of *Actinobacteria*, *Bifidobacteriales*, *Burkholderiales*, *Bacteroidaceae*, *Dorea*, *Fusicatenibacter*, and *Ruminococcus gauvreauii*, while showing higher abundance of *Enterobacterales*, *Erysipelotrichaceae*, *Holdemanella*, and *Alloprevotella (* Göktürk et al., 2024). A two-sample Mendelian randomization analysis showed that Order *Bifidobacteriales*, Family *Bifidobacteriaceae*, and Genus *RuminococcaceaeUCG009* exerted protective effects on IPF, while Genus *Coprococcus2* promoted the development of IPF (Ren Y. et al., 2024). In the pulmonary fibrosis mouse model established by bleomycin injection, both the diversity and richness of gut microbiota were significantly altered (Luo et al., 2025). Xuanfei Baidu Decoction can alleviate bleomycin-induced pulmonary fibrosis by regulating gut microbiota, with AKK being the core bacterium (Jia et al., 2024).

### 2.7 Pulmonary arterial hypertension

Pulmonary arterial hypertension (PAH) is a malignant pulmonary vascular disease, characterized by increased pulmonary vascular resistance, vasoconstriction, and right ventricular hypertrophy, ultimately leading to right heart failure and death (McLaughlin and McGoon, 2006). Researchers are increasingly focusing on the interaction between intestinal dysbiosis and PAH, and the gut microbiota may play an important role in PAH (Chen et al., 2022; Wu P. et al., 2022). A small-scale clinical cohort study showed that in the PAH patient cohort, bacteria producing butyrate and propionate—such as *Coprococcus*, *Butyrivibrio*, *Lachnospiraceae*, *Eubacterium*, *Akkermansia*, and *Bacteroides*—were reduced (Kim et al., 2020). Unlike healthy participants, this may be associated with elevated inflammatory cytokines and endotoxins (Ikubo et al., 2022). Another study found that the relative abundance of *Lachnospiraceae bacterium GAM79* in the feces of PAH patients is lower than that in non-PAH control subjects (Jose et al., 2022). Fecal microbiota transplantation (FMT) can affect PAH phenotypes, and that multi-kingdom markers are more accurate in diagnosing PAH (Chen et al., 2025). Moutsoglou DM et al. also indicated that PAH patients had poor gut microbiome diversity and distinct phylogenetic characteristics, which were manifested by a reduction in anti-inflammatory metabolite-related microorganisms and their products, as well as an enrichment of pro-inflammatory metabolite-related microorganisms (Moutsoglou et al., 2023). Animal experiments have demonstrated that PAH rats exhibit significant changes in intestinal pathology and gut microbiota, as well as increased sympathetic nerve activity (Sharma et al., 2020). Moreover, supplementation of lactobacillus in PAH mice can restructure the intestinal microflora/mycobiome, restore intestinal health, inhibit systemic inflammation, reduce GP130 ligands and related right ventricular cardiomyocyte microtubule remodeling, thereby improving PAH (Prisco et al., 2025).

### 2.8 Acute lower respiratory infection

The microbial diversity of H1N1 patients is reduced, and the number of several representative anaerobic butyrate-producing bacteria from the *Lachnospiraceae* and *Ruminococcaceae* families is significantly decreased (Gu et al., 2020). In infants with bronchiolitis, a significant reduction in intestinal microbial richness and an increase in respiratory microbial diversity can be observed. This phenomenon is also present in infants with the most severe symptoms and those who experience recurrent wheezing episodes after discharge, and is associated with respiratory morbidity (Cabrera-Rubio et al., 2024). Animal experiments have confirmed that in the RSV-infected group, the genus *Aggregatibacter* is enriched while the genus *Proteus* is reduced, resulting in impaired development of intestinal Th17/Treg (Liu J. et al., 2024). Compared with the control group, the gut microbiota of neonates with neonatal acute respiratory distress syndrome (NARDS) undergoes significant changes, and these changes are associated with alterations in lung microbiota, as well as tryptophan metabolites in the lungs and plasma (Yang J. et al., 2024).

### 2.9 Tuberculosis

Tuberculosis (TB) is a highly contagious disease caused by *Mycobacterium tuberculosis*. The occurrence of intestinal dysbiosis in extraintestinal diseases suggests that native intestinal bacteria may affect trans-organ diseases, especially tuberculosis (Lin et al., 2024; Yuan et al., 2024). There is a close association between changes in the intestinal microbiota and *Mycobacterium tuberculosis* infection (Chai et al., 2025). A cross-sectional study showed that in the TB group, dominant genera such as *Bacteroides*, *Parabacteroides*, *Fusobacterium*, and *Lachnoclostridium* were significantly enriched; in contrast, the healthy people group was enriched with *Blautia*, *Roseburia*, *Bifidobacterium*, *unclassified_Ruminococcaceae*, *Clostridium*, and *Romboutsia (* Wang et al., 2022), and certain anaerobic bacteria in feces may be associated with the upregulation of pro-inflammatory immune pathways (Naidoo et al., 2021). At the genus level, compared with the healthy control group, the relative abundance of *Bacteroides*, *Parabacteroides*, and *Veillonella* was significantly higher in the TB group, while the relative abundance of *Faecalibacterium*, *Bifidobacterium*, *Agathobacter*, and *CAG-352* was significantly lower (Ye et al., 2022). A preliminary study in China also confirmed that tuberculosis patients have reduced diversity of intestinal microbiota and lower abundance of short-chain fatty acid-producing genera (Shi et al., 2021), and gut microbial dysbiosis is strongly correlated with changes in IL-17 and IFN-γ (Han et al., 2024). Systematic review and analysis found significant differences in gut microbiota status between the tuberculosis group and healthy controls, characterized by excessive enrichment of *Proteobacteria* and depletion of some short-chain fatty acid-producing bacterial genera such as *Bifidobacteria*, *Roseburia*, and *Ruminococcus*, with anti-tuberculosis treatment exacerbating this dysbiosis (Baral et al., 2023; Li W. et al., 2024). The study found that broad-spectrum antibiotic treatment increases the susceptibility of mice to Mycobacterium tuberculosis, while Bacteroides fragilis can enhance anti-tuberculosis immunity by regulating lncRNA-CGB (which regulates IFN-γ expression through interaction with EZH2), revealing the role of the intestinal bacteria-related axis in tuberculosis immune protection and a new paradigm for treatment (Yang et al., 2022).

## 3 Metabolites of intestinal microbiota

**Figure 2:**
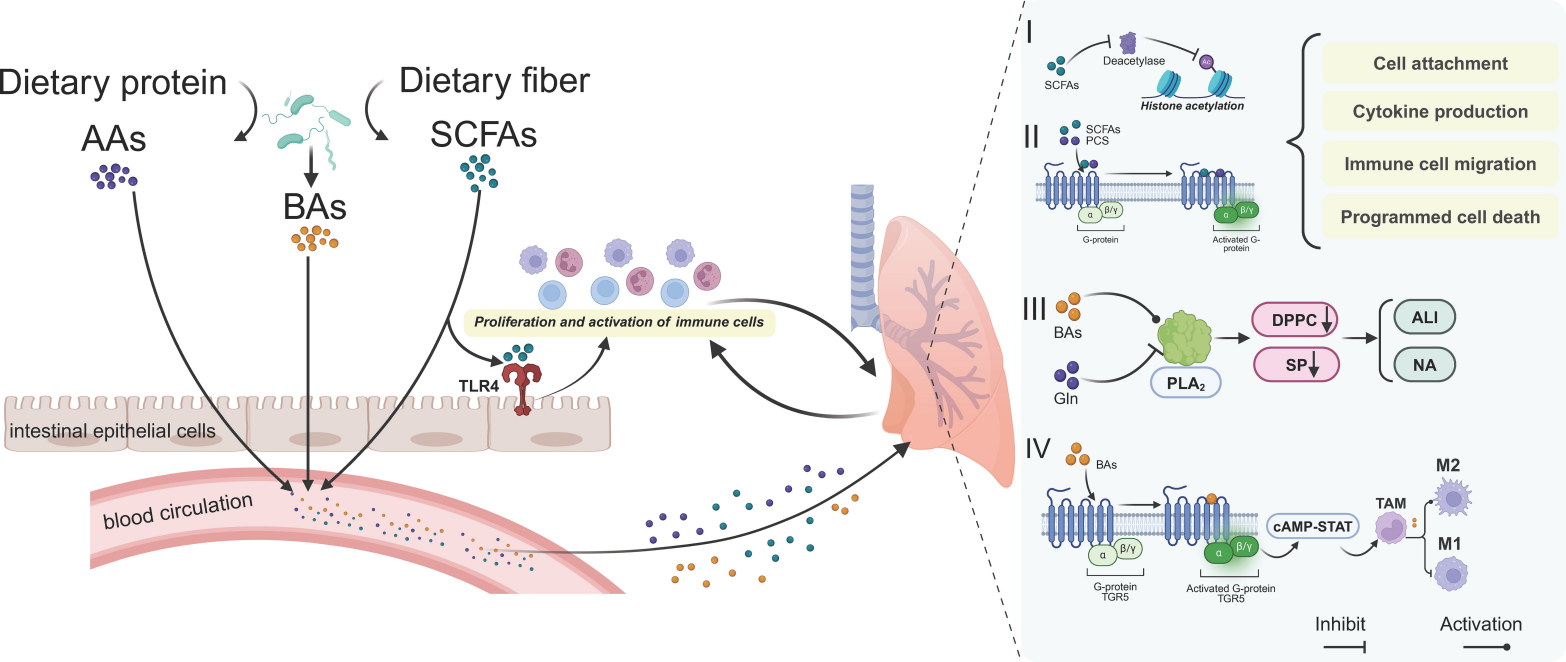
The role of gut microbiota metabolites in pulmonary diseases. Gut microbiota-derived metabolites can affect lung homeostasis through the bloodstream mainly based on four pathways. I) SCFAs act as deacetylase inhibitors and II) activate downstream pathways by binding to G-protein-coupled receptors. Together, they jointly regulate cell adhesion, cytokine production, immune cell migration and programmed cell death, etc. III) Metabolites such as BAs regulate PLA2 to affect pulmonary surfactant proteins and thus influence the progress of ALI and NA diseases. IV) In tumor tissues, metabolites such as BAs affect tumor immunity by regulating the polarization of TAMs. AAs, amino acids; BAs, bile acids; SCFAs, short-chain fatty acids; Gln, glutamine; ALI, acute lung injury; NA, non-eosinophilic asthma; TAM, tumor-associated macrophage.

Metabolites derived from gut microbiota exhibit distinct characteristics in the context of respiratory disease development. Recent advancements in the study of host-microbe interactions have highlighted the critical role of gut microbial metabolites in maintaining tissue and immune homeostasis (Rastogi et al., 2022), as illustrated in **Figure 2**.

### 3.1 Short-chain fatty acids

SCFAs including acetate, propionate and butyrate (Ney et al., 2023) are produced by gut bacteria through the fermentation of indigestible dietary fibers (Liu M. et al., 2024). These metabolites serve as a crucial link between the gut microbiota and the immune system, playing a vital role in maintaining immune homeostasis. Accumulating evidence indicates that SCFAs regulate the activities of various cell types within the body, including but not limited to inhibition of Th1 cells by SCFAs (Che et al., 2024), the effect of SCFAs activate the NLRP3 inflammasome in human macrophages upon TLR stimulation (Wang W. et al., 2024), and it can also effects on the B cells and plasma cells (Mann et al., 2024) and so on. Although the underlying mechanisms are not fully elucidated, G-protein coupled receptor (GPCR) activation and histone deacetylase (HDAC) inhibition are believed to play significant roles (Mann et al., 2024). SCFAs are essential for cellular homeostasis. They are through β-oxidation and regulate macromolecular synthesis, GPCR and HDAC activities, protein modifications, signaling pathways, and gene expression in cells within the tumor microenvironment, particularly in tumor and immune cells (Li S. et al., 2025). As a result, manipulating SCFAs levels by altering gut microbiota composition holds potential for cancer prevention and treatment. Among the components involved in the gut-lung axis, SCFAs are the most important immunomodulatory metabolites, exhibiting protective effects in patients with airway inflammation (Cait et al., 2018). The severe eosinophil inflammation cluster characterized by lower SCFAs levels and higher mucus plug scores (Tanabe et al., 2025). SCFAs enhance the proliferation and activation of immune cells by activating the Toll-like receptor (TLR) signaling pathway in intestinal epithelial cells (Ruan et al., 2022), thereby bolstering the body’s defense against respiratory pathogens. Additionally, SCFAs may induce myelopoiesis and contribute to an anti-inflammatory environment (Trompette et al., 2014), further underscoring their therapeutic potential (Chen et al., 2024). In conclusion, SCFAs help to maintain gut barrier integrity, reduce systemic inflammation and regulate immune responses.

### 3.2 Bile acids

Primary bile acids are produced by the host in the liver to solubilize dietary lipids and fat-soluble vitamins in the small intestine. The primary bile acid pool is largely recycled back to the liver, but a small proportion of these bile acids escapes to the large intestine where they are readily deconjugated and further metabolized by the microbiota into secondary bile acids, which have important effects on the host (Nicolas and Chang, 2019). BAs as critical intestinal metabolites are involved in numerous signaling pathways associated with respiratory diseases (Yin et al., 2023). Yuqiong He et al. verified BAs alleviated sepsis-induced lung injury through blocking PANoptosis-like cell death via STING pathway (He et al., 2023). Furthermore, the signaling effects of nitroolefins can mitigate allergic airway disease by modulating bile acid metabolism (Manni et al., 2021). Additionally, microaspiration of BAs may promote pulmonary fibrosis by stimulating the expression of fibrotic mediators and activating the TGF-β1/Smad3 signaling pathway (Chen et al., 2017). Jose A. Caparrós-Martín et al. investigated the role of bile acids in bronchoalveolar lavage fluid (BALF) in relation to inflammation and microbiota establishment in early cystic fibrosis lung disease (Caparrós-Martín et al., 2023). Secreted phospholipase A2 (sPLA2) as a key enzyme in the acute lung injury pathway (Nakos et al., 2005) is significantly influenced by bile acids. High concentrations of BAs (5 μmol/L) have been shown to markedly increase sPLA2 activity, leading to enhanced surfactant catabolism and exacerbation of acute lung injury (De Luca et al., 2009). Furthermore, the bile acid-responsive G-protein-coupled receptor (TGR5) has been implicated in tumor-associated macrophage (TAM) polarization. TGR5 activation converts TAMs into a tumor-promoting M2-like phenotype by activating the cAMP-STAT3/STAT6 signaling pathway. This mechanism highlights the clinical relevance of TGR5, as its co-expression with high TAM infiltration is significantly associated with prognosis and overall survival in non-small cell lung cancer patients (Zhao et al., 2022).

### 3.3 Tryptophan

Tryptophan is an essential aromatic amino acid acquired through common diet sources and it plays a significant role in respiratory health (Hou et al., 2023). Tryptophan metabolites may modulate asthma through the gut microbiota (Wang H. et al., 2024). Giorgia Renga confirmed that the Tph isoform 1 (Tph1)/5-hydroxytryptamine (5-HT) metabolic pathway of tryptophan plays a key role in regulating pulmonary inflammation (Renga et al., 2024). Tryptophan metabolism in the gut includes the kynurenine (Kyn), 5-HT and indole pathways (Xue et al., 2023), and they have been confirmed to be associated with a variety of inflammatory responses in the lungs. Abbas F Almulla confirmed that the serum Kyn/tryptophan ratio was significantly increased in COVID-19 patients compared to controls (Almulla et al., 2022). Kyn suppresses excessive inflammation by activating the AHR and regulating Th17/Tregs balance (de Araújo et al., 2017). A recent study showed that patients with untreated pulmonary hypertension maintained lower levels of tryptophan and higher concentrations of Kyn compared to controls (Yang C. et al., 2024). Indoleamine 2,3-dioxygenase (IDO) as the enzyme of Tryptophan, reduced IDO activity and expression in sputum of COPD patients is negatively correlated with clinical severity (Naz et al., 2019). During respiratory infection, fungi, bacteria or viruses induce IDO1 expression and enzymatic activity in epithelial, immune or even endothelial cells (Pamart et al., 2024). Xue Lu suggested that 5-HT upregulated airway responsiveness in ovalbumin (OVA)-induced allergic asthma (Lu et al., 2025). 5-HT promotes fibroblast proliferation leading to increased lung fibrosis through activation of TGF-β signaling (Xie et al., 2023). 5-HT activates proliferation (ERK1/2 pathway) and vasoconstriction of pulmonary artery smooth muscle cells via 5-HT2A/2B receptors (Yun and Park, 2025). Yumeng Huang’s study uncovers a mechanism by which peripheral 5-HT aggravated sepsis-induced ALI by promoting neutrophil extracellular trap (NET) formation in the lung of septic mice (Huang et al., 2021).

Based on the above insights, we believe that gut microbial metabolites can have complex regulatory effects on the lungs to a large extent. How to make gut metabolites achieve an immune homeostasis state in regulating lung diseases and its underlying mechanisms still need to be further elaborated in the future studies.

## 4 Interventions based on gut microbiota

**Figure 3:**
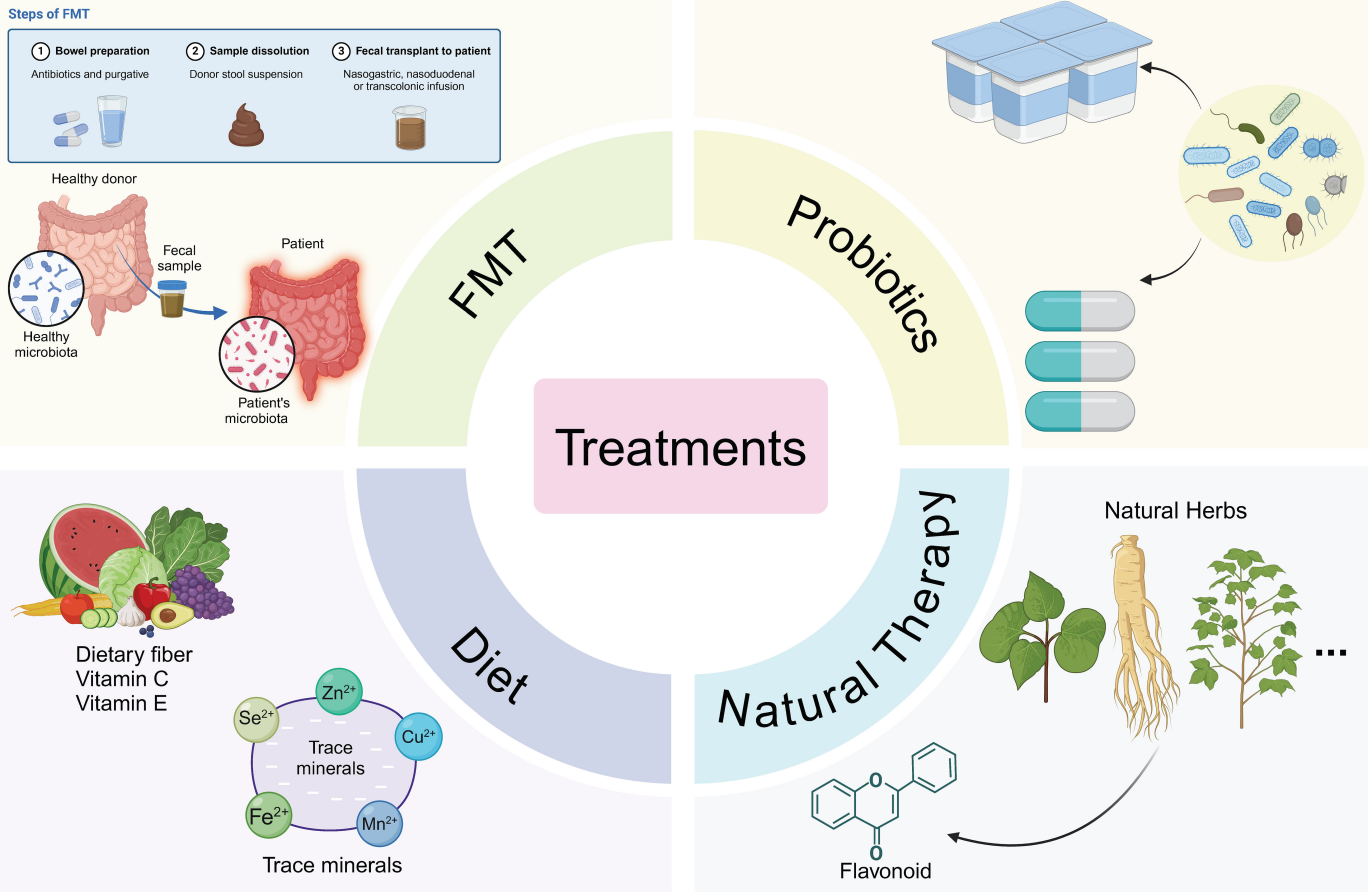
Therapies targeting pulmonary diseases based on the gut microbiome. Fecal microbiota transplantation (FMT) operates by rectifying the dysbiosis of the gut microbiota in patients, thereby fulfilling its therapeutic function with a gut-targeted approach. Administration of probiotic formulations or live-culture-containing yogurts is conducive to ameliorating the pulmonary immune status. Dietary fiber supplementation as well as trace element supplementation also play a part in optimizing the disease condition. Natural botanicals exert their efficacy through anti-inflammatory and antioxidant mechanisms.

As a critical immune barrier for the organism, the gut not only regulates the absorption of nutrients and electrolytes but also plays a pivotal role in preventing the translocation of pathogens and their toxins into the bloodstream. In this review, we specifically highlight the function of microbial metabolites and their essential contributions to maintaining homeostasis and immune tolerance within the organism. With the continuous advancement of technology, accumulating evidence demonstrates that gut-associated microorganisms exert profound effects on immune responses in distal tissues. Expanding our understanding of individual bacterial species and their metabolites will be fundamental to the future development of microbiota-targeted therapies aimed at preventing or treating systemic inflammatory diseases and infections, as illustrated in **Figure 3**.

### 4.1 Fecal microbiota transplantation

Fecal microbiota transplantation (FMT) has garnered significant attention as an innovative therapeutic approach for inflammation and has been extensively investigated in both preclinical and clinical settings. In animal studies, fecal grafts are administered directly via gavage to mice to assess their potential to ameliorate pathophysiological conditions (Li et al., 2021). Accumulating evidence highlights the therapeutic efficacy of FMT in various respiratory diseases. For example, Tim J. Schuijt et al. utilized FMT in a mouse model of gut microbiota depletion and demonstrated that lung bacterial counts, as well as levels of tumor necrosis factor-α (TNF-α) and interleukin-10 (IL-10), returned to normal levels in FMT-colonized mice within six hours following pneumococcal infection (Schuijt et al., 2016). Similarly, Jia Tang’s research revealed that fecal microbiota transplantation inhibits NF-κB phosphorylation and reduces the release of inflammatory cytokines in the lungs, further underscoring its anti-inflammatory potential (Tang et al., 2021). In the context of the recent COVID-19 pandemic, FMT has also been explored as a novel therapeutic strategy. Experimental studies have confirmed its effectiveness in treating COVID-19, offering a promising avenue for managing this disease (Kazemian et al., 2021; Biliński et al., 2022). Collectively, these findings provide robust evidence supporting the role of FMT in treating inflammation-induced infections, highlighting its potential as a valuable intervention for respiratory and systemic inflammatory conditions.

### 4.2 Probiotics

Probiotics are recognized as functional foods that play a significant role in enhancing host health (Lee C. et al., 2024). When combined with prebiotics (indigestible dietary fibers or carbohydrates), they confer additional benefits to the host through anaerobic fermentation (Sasaki et al., 2025). Numerous studies have demonstrated that probiotics have dual effects of immunomodulation and infection inhibition in pulmonary diseases (Bezemer et al., 2024; Hu et al., 2024). For instance, researchers have shown that *Bifidobacterium longum* demonstrated the ability to inhibit the transfer of drug resistance plasmids among Carbapenem-resistant *Klebsiella pneumoniae* (CRKP) strains, thus limiting the horizontal spread of resistance genes (Wang J. et al., 2024). It demonstrated that *Bifidobacterium longum* mitigates CRKP-induced infections. In a multicenter retrospective study, Kazuki Takada et al. demonstrated that probiotic supplementation is associated with improved clinical outcomes in patients with advanced or recurrent non-small cell lung cancer (NSCLC) receiving anti-PD-1 monotherapy (Takada et al., 2021). Additionally, *Lactobacillus rhamnosus* has been shown to penetrate hypoxic tumor regions, enabling efficient delivery of the clustered regularly interspaced short palindromic repeats/CRISPR-associated protein 9 (CRISPR/Cas9) system to tumor sites (Zhong et al., 2023). *Lactobacillus rhamnosus* modulates the STAT signaling-associated immune response to control the lung inflammatory insult and gut dysbiosis in a murine model of asthma-COPD overlap syndrome (ACOS) (Vasconcelos et al., 2025). The relative abundance of AKK, a minor component of the gut microbiota, has been linked to enhanced human responses to anti-PD-1 or anti-PD-L1 immunotherapy (Derosa et al., 2022). Furthermore, studies have reported that airway inflammation and early asthma-like symptoms in COPD mice can be prevented by supplementation with *Bifidobacterium breve* and *Lactobacillus rhamnosus (* Uwaezuoke et al., 2022). Hsin-Chih Lai et al. isolated *Parabacteroides goldsteinii* from the gut microbiota of COPD mice and demonstrated its ability to ameliorate COPD-related inflammation by reducing intestinal inflammation, enhancing mitochondrial and ribosomal activity in colon cells, and restoring aberrant host amino acid metabolism in the serum (Lai et al., 2022). *Bifidobacterium infantis subsp. Infantis YLGB-1496* exhibited good safety and tolerability in young children, and could effectively alleviate gastrointestinal discomfort associated with respiratory diseases (Li P. et al., 2025). In contrast, a trial involving individuals with long-term respiratory symptoms after COVID-19 (without asthma or COPD) showed that after 12 weeks of intervention with *Lactobacillus plantarum GCWB1001*, there was no significant difference in the total score of the primary outcome compared with the placebo group (Kang et al., 2025). The numerical improvements in secondary outcomes were also not statistically significant after correction, and no serious adverse events were reported. Thus, its efficacy required verification in larger-scale trials. Overall, the risk of infection caused by probiotic administration is extremely low, comparable to the risk of infection from commensal bacterial strains. In general, the benefits of probiotic therapy far outweigh the potential risks. Although probiotics are generally proven to be safe, caution must be exercised when using them in specific patient populations, especially those with immunocompromised status (Tsai et al., 2019).

### 4.3 Postbiotics

Postbiotics, together with probiotics, have become widely recognized terms, and interest in them is constantly growing (Swanson et al., 2020; Kumar et al., 2024). As a class of substances with multiple advantages, postbiotics generally exhibit more prominent characteristics compared to probiotics: they have higher safety (Yelin et al., 2019; Vandenplas et al., 2020), usually longer shelf life, and simpler production processes; they pose no risk of antibiotic resistance gene transfer and can be used in immunocompromised populations; they are easy to encapsulate and can be targeted to specific sites (Puccetti et al., 2018), while exerting beneficial effects similar to or even better than those of probiotics (Zhang et al., 2022). In addition, postbiotics also have the feature of being easy to obtain patents, and relevant trials can be conducted in accordance with existing standard pharmacological guidelines by recording pharmacodynamic and pharmacokinetic data (Salminen et al., 2021).

In recent years, postbiotics, as inactivated products and metabolites of probiotics, have shown significant potential in the intervention of various diseases, with their mechanisms closely related to immune regulation and improvement of microecological balance. Studies have demonstrated that extracellular vesicles (EcO83-EVs) from *Escherichia coli A0 34/86* can produce nitric oxide by activating the NF-κB signaling pathway, effectively regulating inflammatory activity both *in vivo* and *in vitro*, thus providing a new direction for replacing live probiotics (Razim et al., 2024). Pasteurized *Weissella cibaria* can improve intestinal mucosal barrier function and reverse gut microbiota dysbiosis (increasing the abundance of anti-inflammatory bacteria and reducing LPS-producing bacteria), thereby improving the survival rate of mice with sepsis-induced ALI and alleviating lung damage (Li Y. et al., 2025).

In the field of cancer treatment, the adjuvant role of postbiotics is particularly prominent. When postbiotic MS-20 is used in combination with anti-PD1 antibodies, it can reshape the tumor immune microenvironment through gut microbiota (increasing effector CD8^+^ T cells and downregulating PD1 expression), inhibiting the growth of colorectal and lung cancers in mice, and the increased abundance of *Ruminococcus bromii* may be involved in this process (Lee PJ. et al., 2024). Although postbiotic JK5G does not significantly improve the objective response rate of advanced non-small cell lung cancer (NSCLC) patients receiving PD-1 inhibitor combined with chemotherapy, it can reduce adverse reactions (such as anemia and nausea) and improve quality of life. Its mechanism is related to regulating gut microbiota, reducing inflammation, and optimizing immune cell subsets, which provides support for enhancing the tolerance of immune checkpoint inhibitor (ICIs) therapy (Chen et al., 2023).

In addition, postbiotics have also shown promising performance in the intervention of inflammatory diseases and oxidative damage. Heat-killed *Lactiplantibacillus plantarum BGPKM22* and its cell-free supernatant exhibit antioxidant activity in bronchial epithelial cells exposed to cigarette smoke, which can exert protective effects by inhibiting the expression of AHR and Nrf2 genes (Babic et al., 2023). Postbiotics from *Saccharomyces cerevisiae* can regulate systemic and mucosal immune responses in calves, enhancing their resistance to bovine respiratory diseases (Maina et al., 2024). Pasteurized yogurt containing heat-killed *Bifidobacterium longum BBMN68* can effectively alleviate mugwort pollen-induced allergic airway inflammation by regulating gut microbiota structure (increasing related beneficial bacteria) and maintaining Th1/Th2 immune balance (Niu et al., 2023).

In summary, different types of postbiotics, through mechanisms such as regulating signaling pathways, improving gut microbiota, and balancing immune responses, show broad application prospects in the fields of inflammation regulation, infection prevention, and adjuvant cancer treatment, providing new ideas for the intervention of related diseases.

### 4.4 Dietary fibers

Dietary influences particularly dietary fiber has long been widely recognized as having a significant impact on the abundance of the human gut microbiota (Sinha et al., 2024). McLoughlin et al. utilized a prebiotic-free inulin formulation to isolate the effects of dietary fiber from those of prebiotics and observed a significant improvement in asthma symptoms in mice after seven days of treatment (McLoughlin et al., 2019). Magdalena Prochazkova et al. conducted a cross-sectional study using multi-omics analyses (16S rRNA sequencing and metabolomics) to compare vegetarians and omnivores, revealing that vegetarians exhibit more favorable glucose and lipid homeostatic profiles. The study also highlighted that increasing vitamin intake, lowering Low-density lipoprotein (LDL) cholesterol levels and limiting the consumption of oxidized lipids can help reduce asthma risk and mitigate symptom exacerbations (Oliver et al., 2021).Flavonoids is an important class of dietary fibers which have also been shown to correlate with the prevalence of chronic respiratory diseases in adults (Wu R. et al., 2024). Resveratrol (RES) is a natural compound produced by many plants in response to stress has been found to induce a dose-dependent upregulation of PD-L1 expression in lung cancer cells. This mechanism is critical for suppressing T-cell-mediated immune responses, as demonstrated in the study (Xin et al., 2024). Furthermore, RES can alleviate asthma symptoms by inducing beneficial microbiota in the gut-lung axis and promoting the normal barrier function of the lungs (Alharris et al., 2022). Isochlorogenic acid C can exert anti-asthmatic effects by promoting the production of SCFAs and other substances by the gut microbiota, regulating immunity, lipid metabolism, and antioxidant responses through the gut-lung axis, and its mechanism is closely related to the gut microbiota (Xu et al., 2025). The use of dietary bioactive compounds, such as phenolic compounds (PC), has emerged as a potential nutritional or therapeutic adjunct for COVID-19 (Augusti et al., 2021). As a representative PC, RES has the potential to serve as a functional supplement to reduce the severity of COVID-19 in patients with poor prognosis due to cardiovascular complications (Gligorijević et al., 2021). A study has shown that supplementation with RES and copper can halve the mortality rate in severe COVID-19 patients (Mittra et al., 2020). In addition, RES acts on the gastrointestinal tract and can alter the gene expression of the gut microbiota (Inchingolo et al., 2022), which may help improve obesity, diabetes and other diseases related to dysbiosis, thereby reducing the impact of COVID-19 and subsequent syndromes.

The research team led by Song et al. found that fermented black barley exerts multiple protective effects through antioxidant activity and regulation of intestinal microbiota: it can not only improve cooking oil fume-induced lung injury in mice, but also correct smoking-induced intestinal dysbiosis by regulating the structure of intestinal microbiota (such as reducing the abundance of *Lactobacillus* and other genera, and increasing the abundance of *Oscillospira* and other genera), thereby alleviating smoking-induced damages such as lung and testis injuries as well as metabolic disorders (Ding et al., 2020; Zhong et al., 2022). Quercetin can effectively regulate intestinal dysbacteriosis, and in particular, significantly increase the abundance of AKK, thereby exerting a protective effect on rats with pulmonary fibrosis (Wu W. et al., 2024). Epigallocatechin gallate (EGCG) from green tea can alleviate obesity-aggravated lung cancer progression by modifying the intestinal microbiome, specifically by increasing the abundance of *Clostridium* and decreasing the abundance of *Deltaproteobacteria* and *Epsilonproteobacteria*, which effect may be closely related to the STAT1/SLC7A11 pathway (Li F. et al., 2023). These findings underscore the profound influence of diet on gut microbiota composition and its subsequent effects on respiratory health, highlighting the potential of dietary interventions in managing chronic respiratory conditions.

## 5 Discussion

Although a wide variety of factors *in vivo* and *in vitro* drive the progression of respiratory diseases (Ma et al., 2022), the balance of gut microorganisms has long been recognized as one of the most significant contributors to or exacerbators of such conditions. The composition and function of the gut microbiota are influenced by numerous intrinsic and extrinsic factors, necessitating further research to elucidate the causes that shape its composition. This article reviews the current state-of-the-art research on respiratory diseases and gut microbes, emphasizing the critical role of the gut-lung axis in respiratory conditions. This includes the composition of gut microbes and the diverse immunomodulatory functions of their metabolites within the organism. However, clinical data on the application of novel therapeutic strategies targeting the gut microbiota remain limited and require further exploration in the future.

## 6 Conclusion

In conclusion, the gut-lung axis plays a pivotal role in the pathogenesis and progression of respiratory diseases, with gut microbiota composition and its metabolites significantly influencing immune responses and disease outcomes. While advancements in multi-omics technologies have deepened our understanding of this relationship, further research is needed to explore the underlying mechanisms and therapeutic potential of targeting the gut microbiota. Strategies such as dietary interventions, probiotics, and FMT hold promise for maintaining gut homeostasis and enhancing immunity, offering novel approaches for the prevention and treatment of respiratory diseases. Future studies should focus on translating these findings into effective clinical applications to improve patient outcomes.
